# The burdens attributable to headache disorders in Cameroon: national estimates from a population-based door-to-door survey, including a headache-care needs assessment

**DOI:** 10.1186/s10194-024-01831-1

**Published:** 2024-08-16

**Authors:** Callixte Kuate Tegueu, Anastase Dzudie Tamdja, Franklin Kom, Blaise Forgwa Barche, Peter Ebasone, Mélanie Magnerou, Paul Mbonda, Yannick Fogang, Daniel Massi Gams, Jacques Doumbe, Andreas Husøy, Timothy J. Steiner

**Affiliations:** 1 Department of Neurology, Douala Laquintinie Hospital, Douala, Cameroon; 2 Faculty of Medicine and Biomedical Sciences, University of Yaoundé, Yaoundé, Cameroon; 3 Department of Internal Medicine, Douala General Hospital, Douala, Cameroon; 4 Clinical Research Education, Networking and Consultancy (CRENC), Yaoundé, Cameroon; 5 Faculty of Medicine and Pharmaceutical Sciences, University of Dschang, Dschang, Cameroon; 6 Department of Neuromedicine and Movement Science, Norwegian University of Science and Technology (NTNU), Edvard Griegs Gate, Trondheim, Norway; 7 Department of Neurology, University of Copenhagen, Copenhagen, Denmark; 8 Division of Brain Sciences, Imperial College London, London, UK

**Keywords:** Headache, Migraine, Tension-type headache, Medication-overuse headache, Epidemiology, Burden, Population-based study, Health-care needs assessment, Cameroon, Sub-Saharan Africa, Global Campaign against Headache

## Abstract

**Background:**

We have previously shown headache to be highly prevalent in Cameroon. Here we present the attributed burden. We also perform a headache-care needs assessment.

**Methods:**

This was a cross-sectional survey among adults (18–65 years) in the general population. Multistage cluster-sampling in four regions (Centre, Littoral, West and Adamawa), home to almost half the country’s population, generated a representative sample. We used the standardised methodology of the Global Campaign against Headache, including the HARDSHIP questionnaire, with diagnostic questions based on ICHD-3 and enquiries into symptom burden, impaired participation (lost productivity and disengagement from social activity), quality of life (QoL) using WHOQoL-8, and willingness to pay (WTP) for effective care. We defined headache care “need” in terms of likelihood of benefit, counting all those with probable medication-overuse headache (pMOH) or other headache on ≥ 15 days/month (H15 +), with migraine on ≥ 3 days/month, or with migraine or tension-type headache (TTH) and meeting either of two criteria: a) proportion of time in ictal state (pTIS) > 3.3% *and* intensity ≥ 2 (moderate-to-severe); or b) ≥ 3 lost days from paid and/or household work in the preceding 3 months.

**Results:**

Among 3,100 participants, mean frequency of any headache was 6.7 days/month, mean duration 13.0 h and mean intensity 2.3 (moderate). Mean pTIS was 9.8%, which (with prevalence factored in) diluted to 6.1–7.4% of all time in the population. Most time was spent with H15 + (5.3% of all time), followed by TTH (1.0%) and migraine (0.8%). For all headache, mean lost days/3 months were 3.4 from paid work, 3.0 from household work and 0.6 from social/leisure activities, diluting to 2.5, 2.2 and 0.6 days/3 months in the population. QoL (no headache: 27.9/40) was adversely impacted by pMOH (25.0) and other H15 + (26.0) but not by migraine (28.0) or TTH (28.0). WTP (maximally XAF 4,462.40 [USD 7.65] per month) was not significantly different between headache types. An estimated 37.0% of adult Cameroonians need headache care.

**Conclusion:**

Headache disorders in Cameroon are not only prevalent but also associated with high attributed burden, with heavily impaired participation. Headache-care needs are very high, but so are the economic costs of not providing care.

## Background

We have recently shown headache disorders to be highly prevalent among adults in Cameroon, with a 1-year prevalence of any headache of 77.1% [1]. The common headache types were tension-type headache (TTH: age- and gender-adjusted prevalence 44.8%) and migraine (18.1%) [1], but a strikingly high proportion of the population (13.1%) reported headache on ≥ 15 days/month (H15 +). About half of these also reported use of acute medication on ≥ 15 days/month, and were considered therefore to have probable medication-overuse headache (pMOH) [1]. Accordingly, the 1-day prevalence of headache (reported headache yesterday [HY]) was also very high (15.3%) [1].

Prevalence is of interest in understanding the global picture of headache disorders, but is not informative of population health. Estimates are required of the health-loss and other burdens attributed to headache. Globally, while TTH is the most prevalent type of headache [2], it is migraine that is associated with greatest lost health at population level [3]. At individual level, important to those affected, higher burdens of lost health and lost productivity are associated with H15 + [4]. It is measures such as these that are relevant to health policy and should guide allocation of limited health resources.

For over two decades the Global Campaign against Headache has supported studies to estimate the burdens attributed to headache disorders in all parts of the world [5], with recent focus on central and western sub-Saharan Africa (SSA) [1, 6], a relatively unploughed field with regard to knowledge of headache. All of these studies have used standardised methodology [7, 8]. Here, using data collected contemporaneously with those on prevalence, we report the burden estimates from the adult general population of Cameroon, and assess the need for headache care in this country.

## Methods

The methodology has been fully documented previously [1], and is briefly described here.

### Ethics

The study was conducted in accordance with the Declaration of Helsinki [9]. Approval was obtained from the Cameroon National Ethics Committee (reference no 2019/04/4458/CE/CNERSH/SP). All participants were informed of the nature and purpose of the study and gave oral consent prior to enrolment.

Data were held in compliance with data-protection regulations.

### Study design

This was a cross-sectional survey employing standardised sampling procedure and questionnaire [7, 8] carried out during June to August 2019. A representative sample of the general population was obtained by multistage cluster-sampling targeting four geographic regions (Centre, Littoral, West and Adamawa) that were home to almost half of the country’s population [10].

Households were randomly chosen within each region and, at visits without prior notice, one adult (aged 18–65 years) was randomly selected from each. Trained interviewers used the structured Headache-Attributed Restriction, Disability, Social Handicap and Impaired Participation (HARDSHIP) questionnaire [7] translated into West-Central African French following the Global Campaign's translation protocol for lay documents [11]. Various modules of HARDSHIP covered multiple aspects of burden, with enquiries addressed to the most bothersome headache type when more than one type was reported.

Responses were entered into a database employing a double-data-entry procedure, with reconciliation of discrepancies by reference to the original questionnaires.

### Analyses

#### Headache diagnoses

These, based on ICHD-3 [12], were made algorithmically [1] in the following order: H15 + (classified as pMOH when associated with acute medication overuse [on ≥ 15 days/month], or otherwise as “other H15 + ”); definite migraine; definite TTH; probable migraine; probable TTH. Definite and probable diagnoses of each were combined in further analyses.

#### Headache-attributed burden

Analyses were made by headache type, and in many cases by gender.

##### Symptom burden

Usual headache intensity, recorded as “not bad,” “quite bad,” and “very bad,” was scaled numerically from 1 to 3. Headache frequency was measured in days/month and usual headache duration in hours. The mean time in ictal state (TIS) for each headache type was derived as a product of attack frequency and duration (capped at 24 h to avoid overestimation), and presented as a proportion of total time (pTIS = TIS/30*24).

##### Lost health

Lost health at individual level was computed for migraine, TTH and pMOH as pTIS*DW, where DW was the disability weight attributed to the ictal state of each disorder (migraine 0.441; TTH 0.037; MOH 0.223 [3, 13]). A DW for other H15 + does not exist.

##### Impaired participation

Impaired participation in complete days over the preceding 3 months was evaluated through the Headache-Attributed Lost Time (HALT) questionnaire [14], incorporated into HARDSHIP [7]. The recognised methodology equated “less than half achieved” to “nothing achieved” and, in counterbalance, “more than half achieved” to “everything achieved” [14]. These losses were assessed separately as losses from income-generating (paid) work, household work and social or leisure activity.

In separate enquiry in those reporting HY, impaired participation in all intended activities yesterday, without distinguishing between work and leisure, was assessed in similar manner as 0% (“less than half” or “nothing” done) or 100% (“more than half” or “everything” done).

##### Overall burden

Two subjective measures were used of overall burden. Quality of life (QoL) was assessed by WHOQoL-8, grading responses to each item from 1 to 5 and generating aggregated scores in the range 8–40, higher scores indicating better QoL. Willingness to pay (WTP) for effective treatment, grounded by the bidding-game method [7], was reported in Central African francs (XAF) per month (in June 2019, USD 1 = XAF 583).

#### Population-level estimates

By factoring in headache prevalence, and adjusting for age and gender, we made estimates of how headache-attributed pTIS and impaired participation (using HALT and HY data) diluted into the general population (those without headache as well as those with).

#### Needs assessment

A needs assessment for headache care counted all those believed likely to benefit from care. We adopted the following criteria: a) having pMOH or other H15 + ; b) having migraine on ≥ 3 days/month; c) having migraine or TTH with pTIS > 3.3% *and* moderate-to-severe headache intensity; d) having migraine or TTH and losing ≥ 3 days in the preceding 3 months from paid and/or household work.

#### Statistics

Descriptive analysis used means with standard errors (SEMs), medians or proportions (%) with 95% confidence intervals (CIs), as appropriate.

Continuous variables were compared using ANOVA-tests (with QoL scores and WTP both treated as though continuous), and categorical variables using chi-squared tests.

Statistical analyses were executed using SPSS version 28 (SPSS, Inc, Chicago, IL). Significance was set at *p* < 0.05.

## Results

A total of 3,100 participants were included. Age- and gender-adjusted 1-year prevalence estimates, reported previously [1] but repeated here because they are required for population-based estimates, were 18.1% for migraine, 44.8% for TTH, 6.5% for pMOH and 6.6% for other H15 + . HY was reported by 15.3%.

### Symptom burden

Overall, mean headache frequency was 6.7 days/month with a mean duration of 13.0 h and mean pTIS of 9.8%. Table 1 shows the symptom burdens attributed to the various headache types.

**Table 1:** Symptom burden, time in ictal state and lost health by headache type and gender

	**Overall**	**Male**	**Female**	**Male vs female**
	**mean ± SEM, median**			
**Frequency** (days/month)				
Any headache	6.7 ± 0.2, 2.0	5.4 ± 0.3, 2.0	7.6 ± 0.3, 3.0	F(1, 2,386) = 34.0, *p* < 0.001
pMOH	27.6 ± 0.4, 30.0	27.4 ± 0.7, 30.0	27.7 ± 0.4, 30.0	F(1, 198) = 0.2, *p* = 0.69
Other H15 +	23.2 ± 0.6, 30.0	23.1 ± 1.0, 30.0	23.2 ± 0.7, 30.0	F(1, 206) = 0.0, *p* = 0.88
Migraine	2.8 ± 0.1, 2.0	2.6 ± 0.1, 2.0	3.0 ± 0.1, 2.0	F(1, 584) = 4.4, *p* = 0.04
TTH	2.8 ± 0.1, 2.0	2.7 ± 0.1, 2.0	2.9 ± 0.1, 2.0	F(1, 1,361) = 1.0, *p* = 0.33
**Duration** (hours)				
Any headache	13.0 ± 0.5, 3.0	11.9 ± 0.8, 2.0	13.9 ± 0.7, 3.0	F(1, 2,305) = 3.4, *p* = 0.06
pMOH	13.7 ± 0.8, 24.0	12.9 ± 1.5, 15.0	13.9 ± 0.9, 24.0	F(1, 197) = 0.3, *p* = 0.57
Other H15 +	9.7 ± 0.7, 4.0	9.2 ± 1.2, 3.0	9.9 ± 0.9, 4.0	F(1, 204) = 0.3, *p* = 0.62
Migraine	19.5 ± 1.2, 4.0	17.6 ± 1.9, 4.0	20.7 ± 1.6, 5.0	F(1, 572) = 1.5, *p* = 0.22
TTH	10.5 ± 0.7, 2.0	10.3 ± 0.9, 2.0	10.7 ± 1.0, 2.0	F(1, 1317) = 0.1, *p* = 0.77
**Intensity** (not bad, quite bad, very bad [n]; means calculated by equating to 1, 2, 3 and treating as though continuous data)				
Any headache	35; 1,487; 802 (mean = 2.3)	16; 685; 316 (mean = 2.3)	19; 802; 486 (mean = 2.4)	*X*^*2*^(2, N = 2,324) = 9.5, *p* = 0.009
pMOH	1; 98; 98 (mean = 2.5)	0; 26; 29 (mean = 2.5)	1; 72; 69 (mean = 2.5)	*X*^*2*^(2, N = 197) = 0.6, *p* = 0.73
Other H15 +	1; 85; 111 (mean = 2.6)	1; 31; 37 (mean = 2.5)	0; 54; 74 (mean = 2.6)	*X*^*2*^(2, N = 197) = 2.1, *p* = 0.36
Migraine	5; 255; 323 (mean = 2.6)	2; 99; 121 (mean = 2.5)	3; 156; 202 (mean = 2.6)	*X*^*2*^(2, N = 583) = 0.1, *p* = 0.94
TTH	28; 1,045; 265 (mean = 2.2)	13; 526; 128 (mean = 2.2)	15; 519; 137 (mean = 2.2)	*X*^*2*^(2, N = 1,338) = 0.5, *p* = 0.79
**Proportion of time in ictal state** (%)				
Any headache	9.8 ± 0.5, 0.8	6.8 ± 0.6, 0.8	12.1 ± 0.7, 1.1	F(1, 2,304) = 29.7, p < 0.001
pMOH	52.4 ± 3.1, 50.0	49.1 ± 6.0, 35.4	53.7 ± 3.7, 63.3	F(1, 197) = 0.4, *p* = 0.51
Other H15 +	31.0 ± 2.5, 11.7	27.9 ± 4.1, 8.3	32.6 ± 3.1, 12.5	F(1, 204) = 0.8, *p* = 0.38
Migraine	4.6 ± 0.3, 1.1	3.9 ± 0.4, 0.8	5.1 ± 0.4, 1.3	F(1, 572) = 3.9, *p* = 0.047
TTH	2.3 ± 0.1, 0.5	1.9 ± 0.2, 0.5	2.7 ± 0.3, 0.4	F(1, 1,316) = 6.5, *p* = 0.01
**Lost health**^a^ (%)				
pMOH	11.7 ± 0.7,11.2	10.9 ± 1.3, 7.9	12.0 ± 0.8, 14.1	F(1, 197) = 0.4, *p* = 0.51
Migraine	2.0 ± 0.1, 0.5	1.7 ± 0.2, 0.4	2.2 ± 0.2, 0.6	F(1, 572) = 3.9, *p* = 0.047
TTH	0.1 ± 0.0, 0.0	0.1 ± 0.0, 0.0	0.1 ± 0.0, 0.0	F(1, 1,316) = 6.5, *p* = 0.01

*pMOH* probable medication-overuse headache, *H15* + headache on ≥ 15 days/month, *TTH* tension-type headache

^a^calculated as pTIS*DW (see text)

For migraine, mean reported headache intensity was 2.5 for males, 2.6 for females, both corresponding to moderate-to-severe pain. Mean headache frequency was 2.8 days/month, significantly higher among females (3.0 days/month) than males (2.6 days/month; *p* = 0.04). Headache duration was not significantly different between genders, but heavily skewed (overall mean 19.5 h, median 4.0 h). Mean pTIS was 4.6% overall, higher among females (5.1%) than males (3.9%; *p* = 0.047), generating individual lost-health estimates of 2.0% (4.6%*0.441) overall, 1.7% (3.9%*0.441) for males and 2.2% (5.1*0.441) for females.

For TTH, mean intensity was 2.2 (moderate pain) in both genders. Mean headache frequency was 2.8 days/month (males 2.7; females 2.9; *p* = 0.33). Headache duration was 10.3 h among males and 10.7 h among females. Mean pTIS was 2.3%, significantly higher in females (2.7%) than males (1.9%; *p* = 0.01). Individual lost health for TTH was 0.1% (2.3*0.037).

There were no gender-related differences in symptom burden for pMOH or other H15 + . Both, inevitably, were associated with much more frequent headache (27.6 and 23.2 days/month respectively). Mean intensities were 2.5 and 2.6, and mean durations 13.7 and 9.7 h. pTIS was 52.4% for pMOH and 31.0% for other H15 + . Individual health loss for pMOH was 11.7% (52.4*0.223).

### Impaired participation

**Fig. 1:**
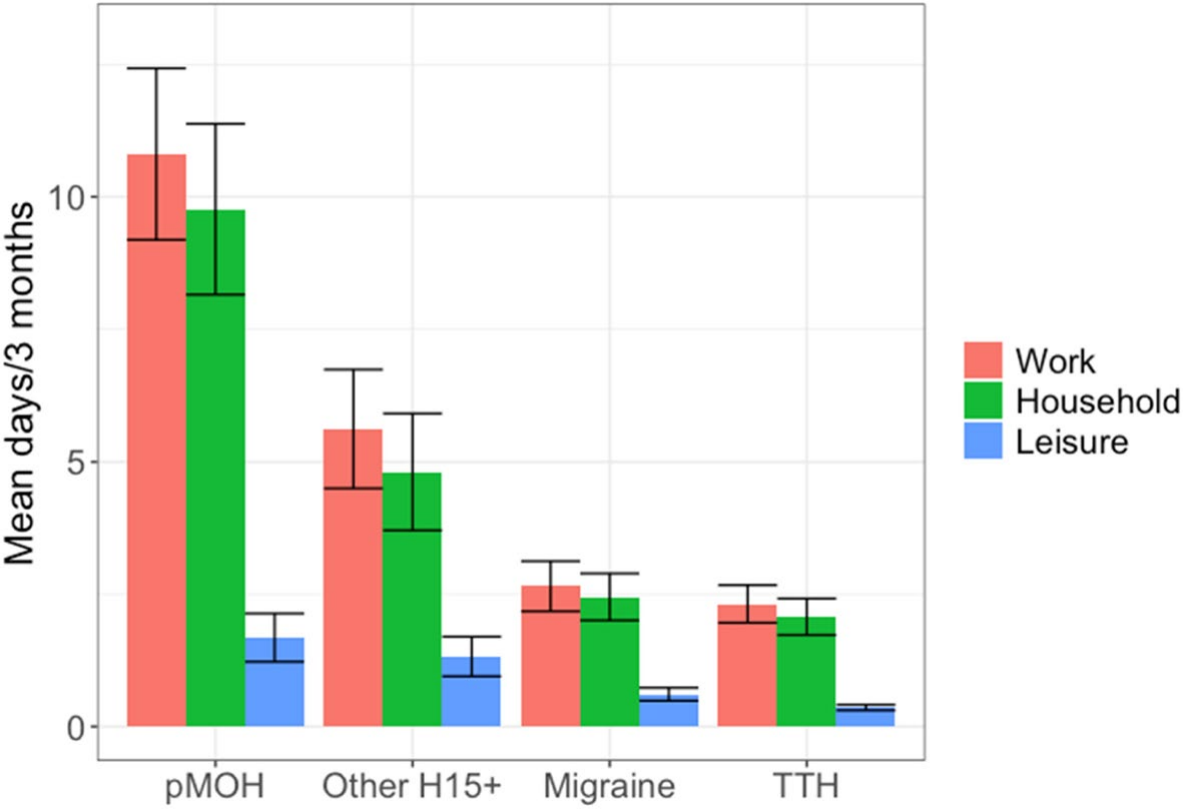
Impaired participation in paid and household work and leisure activity, by headache type (error bars: 95% confidence intervals; pMOH: probable medication-overuse headache; H15 + : headache on ≥ 15 days/month; TTH: tension-type headache)

Table 2 and Fig. 1. show headache-attributed impaired participation. Overall and on average, headache was associated with individual lost time from paid work of 3.4 days/3 months and from household work of 3.0 days/3 months. Both were significantly higher among females than males (paid: 4.0 vs 2.6 days, *p* < 0.001; household: 3.8 vs 2.1 days, *p* < 0.001).

**Table 2:** Impaired participation^a^ by headache type and gender

	**Overall**	**Male**	**Female**	**Male vs female**
	**mean ± SEM, median**			
**Lost time from paid work** (days/3 months)				
Any headache	3.4 ± 0.2, 0.0	2.6 ± 0.2, 0.0	4.0 ± 0.2, 0.0	F(1, 2,367) = 19.3, p < 0.001
pMOH	10.8 ± 0.8, 7.0	10.6 ± 1.6, 6.0	10.9 ± 1.0, 7.0	F(1, 196) = 0.0, *p* = 0.85
Other H15 +	5.6 ± 0.6, 2.0	6.0 ± 0.9, 3.0	5.4 ± 0.7, 2.0	F(1, 205) = 0.3, *p* = 0.61
Migraine	2.7 ± 0.2, 0.0	1.9 ± 0.3, 0.0	3.1 ± 0.3, 0.0	F(1, 581) = 6.4, *p* = 0.01
TTH	2.3 ± 0.2, 0.0 F(3, 2,335) = 89.9, *p* < 0.001	1.9 ± 0.2, 0.0	2.7 ± 0.3, 0.0	F(1, 1,349) = 5.6, *p* = 0.02
**Lost time from household work** (days/3 months)				
Any headache	3.0 ± 0.1, 0.0	2.1 ± 0.2, 0.0	3.8 ± 0.2, 0.0	F(1, 2,364) = 30.8, p < 0.001
pMOH	9.8 ± 0.8, 7.0	9.8 ± 1.6, 3.0	10.1 ± 0.9, 8.0	F(1, 195) = 0.4, *p* = 0.55
Other H15 +	4.8 ± 0.6, 1.0	4.2 ± 1.0, 0.0	5.1 ± 0.7, 2.0	F(1, 205) = 0.5, *p* = 0.47
Migraine	2.4 ± 0.2, 0.0	1.4 ± 0.3, 0.0	3.1 ± 0.1, 1.0	F(1, 579) = 13.4, p < 0.001
TTH	2.1 ± 0.2, 0.0 F(3, 2,332) = 76.2, *p* < 0.001	1.6 ± 0.2, 0.0	2.6 ± 0.3, 0.0	F(1, 1,349) = 8.4, *p* = 0.004
**Lost time from leisure and social activity** (days/3 months)				
Any headache	0.6 ± 0.0, 0.0	0.6 ± 0.0, 0.0	0.6 ± 0.0, 0.0	F(1, 2,360) = 0.0, *p* = 0.98
pMOH	1.7 ± 0.2, 0.0	1.9 ± 0.4, 0.0	1.6 ± 0.3, 0.0	F(1, 195) = 0.2, *p* = 0.64
Other H15 +	1.3 ± 0.2, 0.0	1.7 ± 0.4, 0.0	1.1 ± 0.2, 0.0	F(1, 205) = 2.5, *p* = 0.11
Migraine	0.6 ± 0.1, 0.0	0.6 ± 0.1, 0.0	0.6 ± 0.1, 0.0	F(1, 577) = 0.1, *p* = 0.74
TTH	0.4 ± 0.0, 0.0 F(3, 2,360) = 26.5, *p* < 0.001	0.4 ± 0.0, 0.0	0.3 ± 0.0, 0.0	F(1, 1,348) = 4.2, *p* = 0.04

*pMOH* probable medication-overuse headache, *H15* + headache on ≥ 15 days/month, *TTH* tension-type headache

^a^measured with HALT questionnaire (see text)

These gender differences were seen for both migraine and TTH. Females with migraine lost, on average, 3.1 days/3 months from both paid and household work, men with migraine lost 1.9 workdays (*p* = 0.01) and 1.4 household days (*p* < 0.001). Females with TTH lost 2.7 days from paid work and 2.6 days from household work, men lost 1.9 days from paid work (*p* = 0.02) and 1.6 days from household work (*p* = 0.004).

pMOH was associated with higher losses from paid (10.8 days/3 months) and household work (9.8 days/3 months) than migraine and TTH (all *p* < 0.001). The same was true for other H15 + (5.6 work days and 4.8 household days; all *p* < 0.001). There were no significant gender-related differences for pMOH or other H15 + .

Losses from leisure and social time were substantially lower than from paid or household work (0.6 days/3 months for any headache), with no gender differences. Again, pMOH (1.7 days) was associated with greatest losses, followed by other H15 + (1.3 days), migraine (0.6 days) and TTH (0.4 days).

### Headache yesterday

Reported mean duration of HY was 8.3 h, similar in males (7.9 h) and females (8.5 h; *p* = 0.54), but median values (2.0 h) indicated skewed data (Table 3). Mean intensity was 2.4 on the scale of 1–3 (moderate-to-severe), with no gender-related difference.

**Table 3:** Symptom burden and impaired participation from headache yesterday

	**Overall**	**Male**	**Female**	**Male vs female**
**Duration** (hours)				
Mean ± SEM, median	8.3 ± + .4, 2.0	7.9 ± 0.7, 2.0	8.5 ± 0.6, 2.0	F(1, 472) = 0.4, *p* = 0.54
**Intensity**				
Not bad (n)	3	102	3	*X*^*2*^(2, *N* = 478) = 1.9, *p* = 0.38
Quite bad (n)	302	63	200	
Very bad (n)	173	165	110	
Mean^a^	2.4	2.4	2.3	
**What done**				
Everything (n)	147	50	97	*X*^*2*^(3, 473) = 1.0, *p* = 0.80
More than half (n)	108	37	71	
Less than half (n)	109	41	68	
Nothing (n)	109	34	75	

^a^Equating to 1, 2, 3, and treating as though continuous data

Of the 473 participants reporting HY, 109 (23%) said they could do nothing of their intended activities, 109 (23%) less than half, 108 (22.8%) more than half and 147 (31.1%) said they could do everything as normal (Table 3). Impaired participation with HY was estimated at 46.1%.

### Quality of life and willingness to pay

**Fig. 2:**
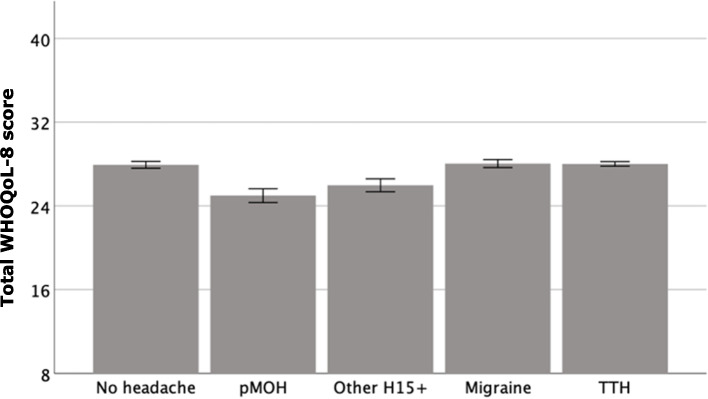
Mean reported quality of life by headache status (assessed using WHOQoL-8 scale, range 8–40; error bars: 95% confidence intervals; pMOH: probable medication-overuse headache; H15 + : headache on ≥ 15 days/month; TTH: tension-type headache)

QoL (no headache: 27.9/40) was reportedly unaffected by migraine (28.0) or TTH (28.0) (Table 4, Fig. 2). In contrast, QoL was significantly impacted by pMOH (25.0) and by other H15 + (26.0) (Fig. 2).

**Table 4:** Quality of life and willingness to pay, by headache status

**Headache status**	**Quality of life**^a^	**Willingness to pay** (XAF/month)
No headache	27.9 ± 0.2, 28.0	-
pMOH	25.0 ± 0.3, 25.0	4,052.10 ± 629.3, 1000.0
Other15 +	26.0 ± 0.3, 26.0	3,675.90 ± 615.1, 1000.0
Migraine	28.0 ± 0.2, 28.0	4,462.40 ± 352, 2000.0
TTH	28.0 ± 0.1, 28.0	3,469.30 ± 764.3, 1000.0
	F(4, 3048) = 30.2, *p* < 0.001	F(3, 2299) = 0.3, *p* = 0.84

*pMOH* probable medication-overuse headache, *H15* + headache on ≥ 15 days/month, *TTH* tension-type headache

^a^Measured with WHO QoL-8

WTP was not significantly different between the headache groups, although those with migraine were reportedly willing to pay more (XAF 4,462.40) than those with pMOH (XAF 4,052.10), who in turn were willing to pay more than those with other H15 + (XAF 3,675.90) or TTH (XAF 3,469.30) (Table 4).

### Population-level estimates

Table 5 shows the proportion of all time (*ie,* among the entire population aged 18–65 years) spent with headache and how this impaired participation at population level. Based on 1-year prevalence and usual headache frequency and duration, an estimated 7.4% of all time was spent with headache. Based on HY data, the estimate was slightly lower (6.1%). Most time was spent with pMOH (3.3%), followed by other H15 + (2.0%), TTH (1.0%) and lastly migraine (0.8%).

**Table 5:** Proportion of time in ictal state and impaired participation at population level, by headache type and by timeframe of enquiry (adjusted for age and gender)

Headache type	Estimated pTIS (%)		Estimated impaired participation			
	**According to 1-year prevalence and mean reported frequency and duration**	**According to prevalence and duration of headache yesterday**	**According to HALT data (lost days/3 months)**			**According to headache yesterday**
			**Lost productivity**		**Lost social or leisure**	**Total impaired participation (%)**
			Paid work	Household work		
Any headache	7.4	6.1	2.5	2.2	0.6	6.9
pMOH	3.3		0.7	0.6	0.1	
Other H15 +	2.0		0.4	0.3	0.1	
Migraine	0.8		0.5	0.4	0.1	
TTH	1.0		1.0	0.9	0.2	

*pTIS* proportion of time in ictal state, *HALT* headache-attributed lost time, *pMOH* probable medication-overuse headache, *H15* + headache on ≥ 15 days/month, *TTH* tension type headache

TTH caused more work days (mean 1.0), household days (0.9) and leisure or social days (0.2) to be lost in the preceding 3 months than any of the other headache types. Overall, headache-attributed lost days/3 months were 2.5 from paid work, 2.2 from household work and 0.6 from leisure or social activity (Table 5). Based on HY data, an estimated 6.9% of all activity was lost to headache.

### Needs assessment

Of the 3,100 participants, 1,167 (37.6%) fulfilled one or more of our criteria for likelihood to benefit from headache care. Table 6 shows the numbers fulfilling each criterion. After adjusting for age and gender, we estimated 37.0% of the adult population of Cameroon needed care. While the largest group were those with H15 + (*n* = 408; adjusted for age and gender: 13.1%), both migraine and TTH also accounted for large numbers, the latter (*n* = 399; 12.8%) more than the former (*n* = 360; 11.3%) (Table 6).

**Table 6:** Headache-care needs assessment

**Criterion fulfilled**		**Proportion of sample**		**Estimated proportion of adult population**^a^
		n	%	% [95% CI]
1	Headache on ≥15 days/month	408	13.2	13.1 [11.9–14.4]
2	Migraine on ≥3 days/month	228	7.4	7.2 [6.3–8.2]
3	Migraine and pTIS >3.3% and moderate-severe intensity	1721	5.5	5.4 [4.6–6.3]
4	Migraine and lost work and/or household days/3 months ≥3	2132	6.9	6.6 [5.8–7.5]
5	TTH and pTIS >3.3% and moderate-severe intensity	192	6.2	6.2 [5.4–7.1]
6	TTH and lost work and/or household days/3 months ≥3	2563	8.3	8.2 [7.3–9.2]
One or more of criteria 1–6		1167	37.6	37.0 [35.3–38.7]

*pTIS* proportion of time in ictal state, *TTH* tension type headache

^a^Age- and gender-adjusted; ^1^of whom 125 also fulfilled criterion 2; ^2^of whom 110 also fulfilled criterion 2, 80 also fulfilled criterion 3 and 62 also fulfilled criteria 2 and 3; ^3^of whom 49 also fulfilled criterion 5

## Discussion

Having shown headache to be highly prevalent among adults in Cameroon [1], here, using data collected contemporaneously from the same participants, we report high levels of burden attributed to it. On average, those reporting any headache in the last year (by definition, an active headache disorder [12]) spend 9.8% of their time with headache (pTIS) of moderate intensity (2.2 on the scale of 0–3). pTIS is, of course, substantially higher among the 13.1% with H15 + (pMOH 52.4%; other H15 + 31.0%) than the 18.1% with migraine (4.6%) or the 44.8% with TTH (2.3%). At population level, a total of 6.1–7.4% of all time is spent having headache, and this is, on average, associated with lost time from income-generating work of 2.5 days/3 months. As an economic indicator of burden, likely to be reflected in national gross domestic product, this is very high indeed (3.8%, assuming a 5-day working week).

Higher productivity losses were seen among those with H15 + than those with migraine or TTH, reflecting the differences in pTIS. Especially, pMOH was associated with estimated losses of 10.8 days/3 months from paid work and 9.8 days/3 months from household work. With its very high prevalence of 6.5% [1], this meant that pMOH contributed more than one quarter (0.7 days/3 months) to all headache-attributed lost income-generating work time (2.5 days/3 months). The importance of this, to health and economic policies, lies in the fact that MOH is an avoidable illness, although avoidance requires public education.

But the impact of TTH on lost productivity is noteworthy: two-fold that of migraine (1.0 vs 0.5 work days, and 0.9 vs 0.4 household days). This is because of its high prevalence: pTIS for TTH was lower than for migraine (owing to shorter headache duration), while estimated lost health from TTH was substantially lower, reflecting both the lower pTIS and the much lower DW (0.037 compared with 0.441 [3, 13]). (Here it should be noted again that DWs, as used by the Global Burden of Disease study [3], are a broad measure of lost health rather than disability [15, 16]). The mean reported headache intensity was indeed higher in migraine than in TTH (2.6 vs 2.2 on the scale 1–3, which may indicate severe vs moderate), but by far less than the difference in DWs. While lost productivity might be expected to correlate with symptom burden (more severe headache leading to greater lost productivity), our earlier study has shown headache *frequency* to be the main driver of lost productivity [17], intensity less so and duration having virtually no impact. Since mean headache frequency was reportedly the same in TTH and migraine (2.8 days/month), the two-fold higher lost worktime for TTH can be well explained by its more than two-fold prevalence (44.8% vs 18.1%) – but this calls for a rethinking of TTH as a generally non-disabling headache [18]. It is possible that, among those diagnosed as TTH, some in fact had migraine. This would have increased the prevalence estimate for TTH while reducing that for migraine, and elevated the burden attributed to TTH. However, the prevalence estimates were very close to those in nearby Benin (TTH 43.1%, migraine 21.2% [6]), suggesting any such error was small. The key message here is that, in Cameroon, although TTH may be less burdensome at individual level than other headache types, its impact at population level is not to be overlooked in health and economic policies.

We saw no significant gender-related differences in frequency, duration, pTIS or impaired participation in paid or household work or leisure activities with regard to H15 + . Migraine, on the other hand, was associated with higher headache frequency, and consequently higher pTIS, among females than among males, and, probably consequentially (wholly or partly), with greater productivity losses from both paid and household work among females than among males. A similar pattern regarding pTIS and lost productivity was seen for TTH, even though mean headache frequency and duration were similar in the two genders (a reflection of skewed data).

With the inclusion of enquiry into HY, and factoring in its prevalence, we had two sets of data for estimating symptom burden and impaired participation. Although HY data were inevitably based on a lower N, they were presumably free from recall error. We have already demonstrated that predicted 1-day headache prevalence (estimates made from 1-year prevalence and reported frequency) tends to be lower than observed 1-day prevalence (*ie*, of HY) [1]. This finding suggests recall underestimates frequency. Here, since we had information on duration of HY, we were also able to calculate pTIS solely from HY data, but, somewhat surprisingly, this gave a slightly lower estimate than the calculation based on headache frequency in days/month and usual headache duration (6.1% vs 7.4%). Recall perhaps overestimates duration. Nevertheless, these two independent calculations corroborated each other in showing that a very substantial proportion of all time in Cameroon is spent having headache.

In contrast, direct comparison between impaired participation calculated from HALT and HY data is not straightforward. Importantly, our method of calculating impaired participation from HY (counting less than half as nothing done, and more than half as everything) has not been validated in the same way as for HALT [14]. It does not make the same distinction between absenteeism from and reduced productivity with headache while at work, but, rather, accepts respondents’ subjective estimates of their actual overall activity yesterday in relation to their intended activity. Furthermore, it does not differentiate between different domains of participation. Still, the estimated 6.9% impairment in participation derived from HY is very high, and underpins our finding (based on HALT) that headache leads to very substantial losses in productivity.

High levels of headache-attributed impaired participation were also reflected in the extremely high proportion of Cameroon’s adult population believed to be in need of headache-care (37.0% when adjusted for age and gender). H15 + (13.1%) is the leading call on headache care, but TTH follows closely: with an estimated 8.2% of the population losing ≥ 3 work and/or household days/3 months from TTH, age- and gender-corrected estimates showed 12.8% of the population to be in need of headache care for this very neglected disorder. A somewhat lower but far from insubstantial 11.3% require headache care for migraine.

The data on QoL deserve comment. WHOQoL-8 is a generally insensitive measure, without intuitively meaningful units, but it showed significantly lower QoL among those with H15 + than among those with migraine, TTH or no headache. High individual symptom burden in H15 + is the most likely explanation, although, despite the symptom burdens associated with migraine and TTH, WHOQoL scores for these did not differ from those for no headache.

WTP was descriptively highest in migraine, without statistical significance. It is a highly subjective measure, with no means of assessing veracity. The large differences between means and medians also demonstrated that the data were heavily skewed.

### Strengths and limitations

An account of the study strengths and limitations has been given previously [1], but is repeated here. The study used established methodology, generated a large sample representative of the country, and put quality-control measures in place. But, as in all such cross-sectional studies, there was dependence on recall, with diagnoses based solely on responses to a diagnostic question set. With a lack of resources (in particular, lack of headache specialists) to validate this question set directly in the population of interest and in the local translation, we depended on its use previously in 20 countries and almost as many languages, with direct validation in four [19–22].

## Conclusion

Headache disorders in Cameroon are not only prevalent but also associated with high attributable burden. With a total of 6.1–7.4% of all time in the population spent having headache, lost time from income-generating work (2.5 days/3 months) is an indicator of high national as well as individual economic burden. H15 + contributes very substantially; pMOH, an avoidable illness, accounts for more than a quarter of lost work time. TTH because of its prevalence in Cameroon, is also a major contributor. An estimated 37.0% of the adult population of Cameroon have need for headache care. This is very high, but so are the economic costs of not providing care.

## Data Availability

The original data are held at Clinical Research Education, Networking and Consultancy (CRENC), Yaoundé, Cameroon, and the analytical set at Norwegian University of Science and Technology, Trondheim, Norway. When analyses are completed, anonymised data will be available on request for academic purposes, in line with the policy of the Global Campaign against Headache.

## Abbreviations

CI: Confidence interval
d/m: Days/month
GBD: Global Burden of Disease
HALT: Headache-Attributed Lost Time (index)
HARDSHIP: Headache-Attributed Restriction, Disability, Social Handicap and Impaired Participation (questionnaire)
HY: Headache yesterday
ICHD: International Classification of Headache Disorders
LTB: *Lifting* *The Burden*
MOH: Medication-overuse headache
pMOH: Probable MOH
pTIS: Proportion of time in ictal state
QoL: Quality of life
SSA: Sub-Saharan Africa
TIS: Time in ictal state
TTH: Tension-type headache
USD: United States dollar
WHOQoL-8: World Health Organization quality of life (8-item questionnaire)
WTP: Willingness to pay
XAF: Central African franc

## References

[1] Kuate Tegueu C, Dzudie Tamdja A, Kom F, Forgwa Barche B, Ebasone P, Magnerou M et al (2024) Headache in the adult population of Cameroon: prevalence estimates and demographic associations from a cross-sectional nationwide population-based study. J Headache Pain 25:42. 10.1186/s10194-024-01748-9 38515027 10.1186/s10194-024-01748-9 PMC10956204

[2] Stovner LJ, Hagen K, Linde M, Steiner TJ (2022) The global prevalence of headache: an update, with analysis of the influences of methodological factors on prevalence estimates. J Headache Pain 23:34. 10.1186/s10194-022-01402-2 35410119 10.1186/s10194-022-01402-2 PMC9004186

[3] Vos T, Lim SS, Abbafati C, Abbas KM, Abbasi M, Abbasifard M et al (2020) Global burden of 369 diseases and injuries in 204 countries and territories, 1990–2019: a systematic analysis for the Global Burden of Disease Study 2019. The Lancet 396:1204–1222. 10.1016/S0140-6736(20)30925-9 10.1016/S0140-6736(20)30925-9 PMC7567026 33069326

[4] Steiner T, Stovner L (2019) Societal impact of headache: Burden, costs and response. Springer Nature, Cham

[5] Steiner TJ, Birbeck GL, Jensen RH, Martelletti P, Stovner LJ, Uluduz D et al (2022) The Global Campaign turns 18: a brief review of its activities and achievements. J Headache Pain 23:49. 10.1186/s10194-022-01420-0 35448941 10.1186/s10194-022-01420-0 PMC9022610

[6] Adoukonou T, Agbetou M, Dettin E, Kossi O, Husøy A, Thomas H, Houinato D, Steiner TJ (2024) The prevalence and demographic associations of headache in the adult population of Benin: a cross-sectional population-based study. J Headache Pain 25:52 38580904 10.1186/s10194-024-01760-z PMC10996250

[7] Steiner TJ, Gururaj G, Andrée C, Katsarava Z, Ayzenberg I, Yu SY et al (2014) Diagnosis, prevalence estimation and burden measurement in population surveys of headache: presenting the HARDSHIP questionnaire. J Headache Pain 15:3. 10.1186/1129-2377-15-3 24400999 10.1186/1129-2377-15-3 PMC3906903

[8] Stovner LJ, Al Jumah M, Birbeck GL, Gururaj G, Jensen R, Katsarava Z et al (2014) The methodology of population surveys of headache prevalence, burden and cost: principles and recommendations from the Global Campaign against Headache. J Headache Pain 15:5. 10.1186/1129-2377-15-5 24467862 10.1186/1129-2377-15-5 PMC3907133

[9] Association WM (2013) World medical association declaration of helsinki: ethical principles for medical research involving human subjects. JAMA 310:2191–2194. 10.1001/jama.2013.281053 24141714 10.1001/jama.2013.281053

[10] Central Bureau of the Census and Population Studies, Cameroon. 3ème RGPH: Population en chiffre. www.bucrep.cm/index.php.fr/recensements/3eme-rgph. Accessed 5 Aug 2023

[11] Peters MBJ, Houchin C, Kandoura T, Steiner TJ (2007) Translation protocol for lay documents. J Headache Pain 8:S43–S44

[12] Headache Classification Committee of the International Headache Society (IHS) (2018) The international classification of headache disorders, 3rd edition. Cephalalgia 38:1–211. 10.1177/0333102417738202 10.1177/0333102417738202 29368949

[13] Salomon JA, Haagsma JA, Davis A, de Noordhout CM, Polinder S, Havelaar AH et al (2015) Disability weights for the global burden of disease 2013 study. Lancet Glob Health 3:e712–e723. 10.1016/s2214-109x(15)00069-8 26475018 10.1016/s2214-109x(15)00069-8

[14] Steiner TJ, Lipton RB (2018) The Headache-Attributed Lost Time (HALT) Indices: measures of burden for clinical management and population-based research. J Headache Pain 19:12. 10.1186/s10194-018-0837-3 29396646 10.1186/s10194-018-0837-3 PMC5796955

[15] Murray CJ, Ezzati M, Flaxman AD, Lim S, Lozano R, Michaud C et al (2012) GBD 2010: design, definitions, and metrics. Lancet 380:2063–2066. 10.1016/s0140-6736(12)61899-6 23245602 10.1016/s0140-6736(12)61899-6

[16] Steiner TJ, Terwindt GM, Katsarava Z, Pozo-Rosich P, Gantenbein AR, Roche SL et al (2022) Migraine-attributed burden, impact and disability, and migraine-impacted quality of life: expert consensus on definitions from a Delphi process. Cephalalgia 42:1387–1396. 10.1177/03331024221110102 35791285 10.1177/03331024221110102 PMC9638708

[17] Husøy A, Katsarava Z, Steiner TJ (2023) The relationship between headache-attributed disability and lost productivity: 3 attack frequency is the dominating variable. J Headache Pain 24:7. 10.1186/s10194-023-01546-9 36782131 10.1186/s10194-023-01546-9 PMC9926851

[18] Steiner T, Lange R, Voelker M (2003) Episodic tension-type headache (ETTH): evidence of prolonged disability from a placebo-controlled comparison of aspirin and paracetamol. Cephalalgia 23:630 10.1046/j.1468-2982.2003.00470.x 12534583

[19] Ayzenberg I, Katsarava Z, Mathalikov R, Chernysh M, Osipova V, Tabeeva G et al (2011) The burden of headache in Russia: validation of the diagnostic questionnaire in a population-based sample. Eur J Neurol 18:454–459. 10.1111/j.1468-1331.2010.03177.x 20722704 10.1111/j.1468-1331.2010.03177.x

[20] Rao GN, Kulkarni GB, Gururaj G, Rajesh K, Subbakrishna DK, Steiner TJ et al (2012) The burden of headache disorders in India: methodology and questionnaire validation for a community-based survey in Karnataka State. J Headache Pain 13:543–550. 10.1007/s10194-012-0474-1 22911168 10.1007/s10194-012-0474-1 PMC3444540

[21] Yu SY, Cao XT, Zhao G, Yang XS, Qiao XY, Fang YN et al (2011) The burden of headache in China: validation of diagnostic questionnaire for a population-based survey. J Headache Pain 12:141–146. 10.1007/s10194-011-0336-2 21452008 10.1007/s10194-011-0336-2 PMC3072517

[22] Herekar AD, Herekar AA, Ahmad A, Uqaili UL, Ahmed B, Effendi J et al (2013) The burden of headache disorders in Pakistan: methodology of a population-based nationwide study, and questionnaire validation. J Headache Pain 14:73. 10.1186/1129-2377-14-73 23967900 10.1186/1129-2377-14-73 PMC3765424

